# *Brucella suis* Infection in Cardiac Implantable Device of Man Exposed to Feral Swine Meat, Florida, USA

**DOI:** 10.3201/eid3104.241721

**Published:** 2025-04

**Authors:** Jose A. Rodriguez, Candice Joseph, Asmita A. Gupte, Alaina S. Ritter, Ramil Goel, Mark Panna, Diansy Zincke, Michael H. Norris, Jason K. Blackburn, Cody B. Barfield, Devin M. Frison, Philip A. Lee, Danielle R. Stanek, Grishma A. Kharod, Elke Saile, Rebekah V. Tiller, Maria E. Negrón, Norman L. Beatty

**Affiliations:** University of Florida College of Medicine, Gainesville, Florida, USA (J.A. Rodriguez, C. Joseph, A.A. Gupte, A.S. Ritter, N.L. Beatty); Malcom Randall Veterans Affairs Medical Center, Gainesville (R. Goel, M. Panna); University of Florida Emerging Pathogens Institute, Gainesville (D. Zincke, J.K. Blackburn); University of Hawai'i at Mānoa, Honolulu, Hawaii, USA (M.H. Norris); Florida Department of Health, Tallahassee, Florida, USA (C.B. Barfield, D. Frison, P.A. Lee, D.R. Stanek); Centers for Disease Control and Prevention, Atlanta, Georgia, USA (G.A. Kharod, E. Saile, R.V. Tiller, M.E. Negrón)

**Keywords:** brucellosis, bacteria, *Brucella suis*, cardiac implantable electronic device, feral swine, *Brucella anthropi*, *Ochrobactrum*, Florida, United States

## Abstract

*Brucella suis* infection in the United States is typically from feral swine exposure. We describe a case of *B. suis* cardiac implantable device infection in a man exposed to meat and blood from feral swine in Florida, USA. The infection was diagnosed using culture, molecular diagnostics, and whole-genome sequencing.

Multiple microorganisms cause cardiac implantable electronic device (CIED) infections, including *Brucella* (*1*,*2*). Brucellosis-causing species of *Brucella* are aerobic, non–spore-forming, gram-negative coccobacilli. *Brucella suis* is 1 of 4 species most known to cause brucellosis, the others being *B. melitensis*, *B. canis*, and *B. abortus* (*3*). We describe a case of *B. suis* CIED infection in Florida, USA, that illustrates diagnostic challenges posed by chronic brucellosis.

## The Study

A 77-year-old man sought treatment at Malcom Randall Veterans Affairs Medical Center, Gainesville, Florida, USA, in fall 2020 with recurrent left chest discomfort. His medical history included controlled type 2 diabetes mellitus, hypertension, dyslipidemia, nonischemic heart failure with reduced ejection fraction (25%–30% [reference range 55%–70%]), and placement of a biventricular automated implantable cardiac defibrillator (AICD) in a prepectoral location. The man’s surgical history was notable for multiple automated cardiac devices and revisions, most recently in 2018 with generator exchange. The man lived on a rural farm in Florida and worked as a pastor. Several reportedly healthy outdoor dogs and 5–10 goats lived on the property, but the patient did not care for or interact with them. The man denied hunting, consuming products from the residential goats, or receiving animal scratches or bites.

The man had multiple hospital admissions for left chest induration, edema, and pain starting in spring 2019. Over the following year, he received multiple courses of antibiotics for culture-negative CIED infection, including 2 weeks of vancomycin and aztreonam, 2 weeks of daptomycin and aztreonam followed by 4 weeks of oral doxycycline and ciprofloxacin, and 2 weeks of daptomycin and ceftriaxone followed by suppression with oral doxycycline and cefdinir. He tolerated suppression for 6 months but discontinued therapy because of side effects. Discussions with the facility’s cardiothoracic surgery team resulted in retention of the man’s CIED. Transesophageal echocardiograms and blood cultures were repeatedly negative.

The man did well for several months after stopping antibiotics but then sought treatment at an outside hospital in Alabama, USA, for left chest discomfort, subjective low-grade fever, and migration of his AICD to the left chest wall below the nipple. A transthoracic echocardiogram showed no vegetations, and blood cultures obtained at admission were initially negative. Physicians discharged the man with no prescribed course of antibiotics. Blood cultures subsequently demonstrated *Ochrobactrum anthropic* which physicians identified using a VITEK 2 microbial identification system (bioMérieux, https://www.biomerieux.com). The patient visited the Gainesville facility in fall 2020 for further workup.

**Figure 1:**
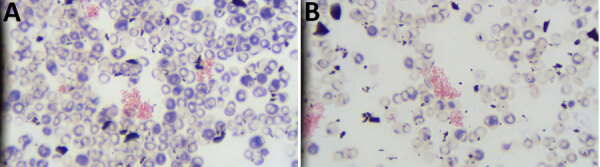
Gram stains of whole blood from study of *Brucella suis* infection in cardiac implantable device of man exposed to feral swine meat, Florida, USA. A) Pink clumps of gram-negative coccobacilli are visible. Original magnification ×400. B) Enlarged area from panel A showing closer view of clumps. Original magnification ×400.

We conducted repeat blood culture tests that yielded growth in 2 of 4 bottles (both aerobic), which could not be speciated further at our laboratory. Gram stain revealed clumps of small, gram-negative coccobacilli (Figure 1). We sent the isolate to reference laboratories, including the Florida Department of Health, Centers for Disease Control and Prevention (CDC), and University of Florida Emerging Pathogens Institute.

**Figure 2:**
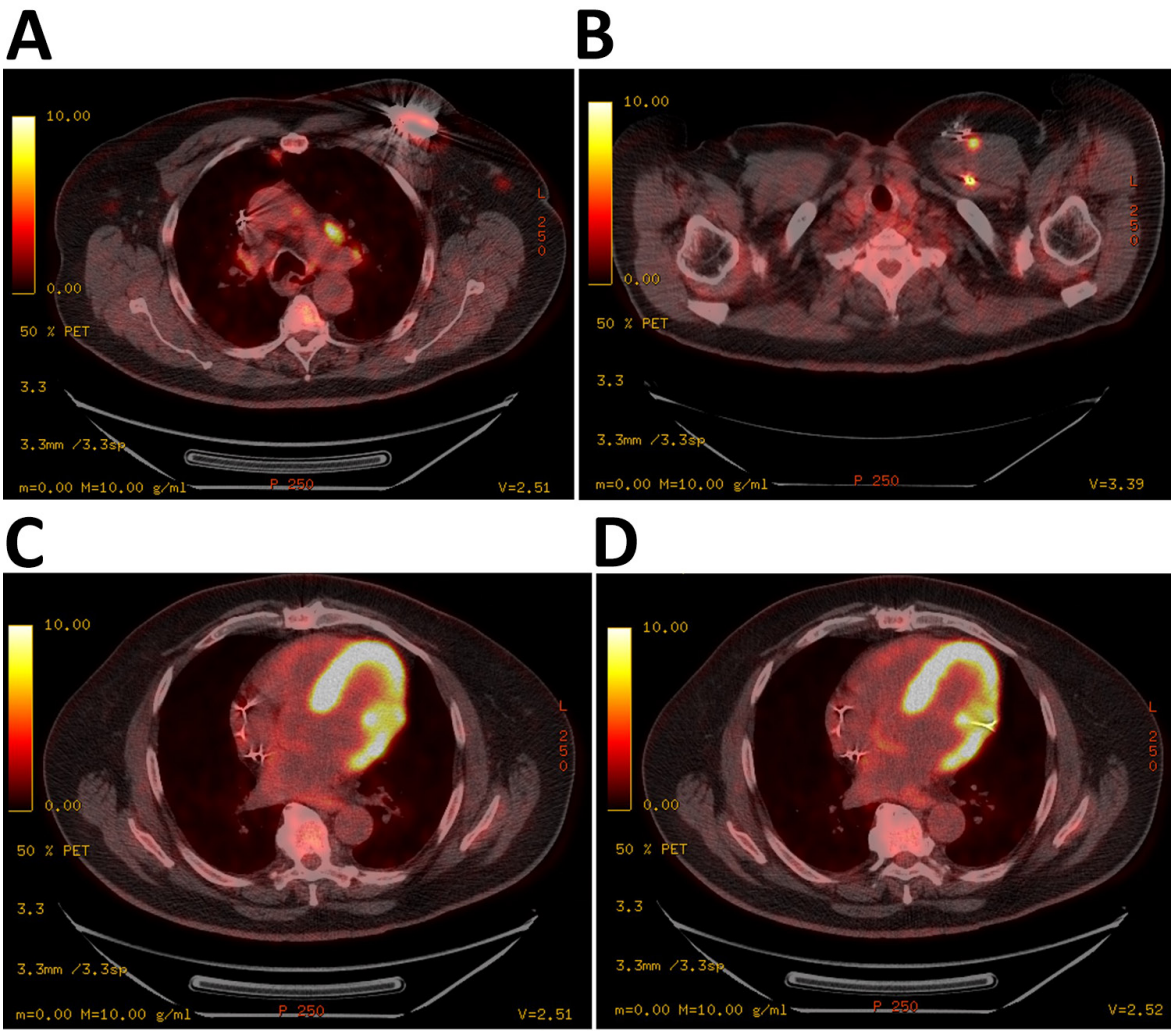
Positron emission tomography scan images from study of *Brucella suis* infection in cardiac implantable device of man exposed to feral swine meat, Florida, USA. A, B) Hypermetabolic activity consistent with infection surrounding the automated implantable cardiac defibrillator generator device (A) and tracking along each generator wire (B). C, D) The infection passes through the chest wall, left subclavian vein, and myocardium into the left ventricle (C) and at the site of implantation of the lead (D).

Transesophageal echocardiogram showed no evidence of vegetation on the valves or AICD leads. An inpatient fluorodeoxyglucose positron emission tomography scan showed increased fluorodeoxyglucose avidity of the generator pocket, surrounding soft tissue, and AICD leads in the left chest wall (Figure 2). We noted no evidence of systemic embolization. Given our concern for the patient’s infection, we removed the AICD. We visualized no clear vegetations on the leads. We sent the device and pocket fluid to the Florida Department of Health and CDC for molecular testing. *Brucella* antibody serology testing (ELISA; Quest Diagnostics, https://www.questdiagnostics.com) returned a positive result of 4.5 for IgG and a negative result of 0.66 for IgM (normal, <0.8; equivocal, 0.8–1.09; positive, ≥1.10). *Brucella* confirmation and species determination took place from clinical specimens (blood, pocket tissue, and device) and cultured isolates. Laboratory technicians used small nucleotide polymorphism PCR, phenotypic analysis for motility, whole-genome sequencing and analysis (GenBank accession no. PRJNA944102), multilocus variable-number tandem-repeat analysis (Appendix), and Laboratory Response Network confirmatory methods to reveal results that were consistent with *B. suis* biovar 1 (*4*).

After *B. suis* identification, we reviewed with the patient any potential exposures to feral swine. He confirmed no hunting activities but recalled receiving feral swine meat as a gift from a local hunter around 2017 on several occasions. He did not remember who specifically gave him the meat but recalled handling the raw meat and blood with bare hands before cooking and consuming it. This encounter likely served as his exposure to *B. suis*. Although the goats and dogs on his property could potentially have served as vectors, the man did not interact with them, and the animals were never tested for brucellosis.

We notified hospital epidemiologists and the Florida Department of Health of the diagnosis, along with the Alabama Department of Health. Given the well-recognized potential risk for infection in exposed laboratory personnel and healthcare providers, we conducted interviews with exposed hospital and laboratory staff employees to identify the risk level of the exposure, per CDC risk stratification standards (*5*). We considered the patient’s surgery to be low risk of generating aerosols, and all operating room staff adhered to requirements for personal protective equipment (gloves, gowns, and masks). Of the laboratory personnel working with the specimens, all of whom wore a laboratory coat, mask, and gloves, 3 had minimal to low-risk exposures and 3 had higher-risk exposures. Employees with higher-risk exposure underwent clinical and serologic monitoring for 6 months and received postexposure prophylaxis as appropriate; no subsequent evidence of brucellosis emerged.

After establishing a diagnosis, we treated the patient with oral doxycycline (100 mg/12 h) and rifampin (300 mg 1×/d) for a total duration of 6 weeks. At the end of his antibiotic course, the patient’s repeat blood cultures remained negative. Four months after device removal, the patient underwent re-implantation of a new AICD on the contralateral side. Approximately 1 year after device removal, a total *Brucella* microagglutination titer performed at CDC was 40 (presumptive positive, ≥160; inconclusive, <160–≥20; negative, <20). On routine outpatient follow-up at >3 years, we noted no clinical evidence of brucellosis.

## Conclusions

In the United States, feral swine (*Sus scrofa*) serve as the principal reservoir for *B. suis*. More than 1 million feral swine live in Florida and can carry zoonoses that include leptospirosis, trichinella, and toxoplasmosis. Feral swine hunters are at risk of contracting *B. suis*, and measures to reduce that risk include the use of personal protective equipment and thoroughly cooking animal products before consumption (*6*). Exposed dogs and goats can also transmit this infection to humans (*7*). One study from an endemic area in the Middle East reported *Brucella* as the causative organism in 11% of CIED infections (*2*), but *Brucella* CIED infections are considered uncommon in the United States. Our case therefore raises awareness of this organism in endemic parts of the United States. It is of diagnostic significance that *B. suis* in our patient’s blood culture was initially misidentified as *O. anthropi*. The VITEK 2 GN (gram-negative) ID card (bioMérieux) can identify various gram-negative bacterial pathogens, including *B. melitensis* and *O. anthropi*. Other VITEK 2 misidentifications have been reported because of similar phenotypic traits between *Brucella* and *Ochrobactrum* species (*8*,*9*). 

CDC recommends a minimum 6- to 8-week treatment course of doxycycline and rifampin or other appropriate dual-drug regimen for acute brucellosis (*5*,*10*). Substantial delays between *Brucella* exposure and clinical symptoms have been previously reported in patients with CIED infections (*11*). In this case, the intermittent use of antibiotics with device retainment likely led to a prolonged clinical course. The combination of source control (device removal) and targeted therapy (doxycycline and rifampin) successfully treated this patient’s *B. suis* CIED infection, with no evidence of infection relapse at >3 years after surgery.

Appendix Additional information for *Brucella suis* infection in cardiac implantable device of man exposed to feral swine meat, Florida, USA.
