# Supplementary material for: Brucella suis Infection in Cardiac Implantable Device of Man Exposed to Feral Swine Meat, Florida, USA
Source: Emerg Infect Dis. 2025 Apr;31(4):795–8. doi: 10.3201/eid3104.241721 (PMC11950250; doi:10.3201/eid3104.241721)
Supplement: Appendix — Additional information for Brucella suis infection in cardiac implantable device of man exposed to feral swine meat, Florida, USA. [file 24-1721-Techapp-s1.pdf]

Article DOI: http://doi.org/10.3201/eid3104.241721
EID cannot ensure accessibility for supplementary materials supplied by authors. Readers who have difficulty accessing supplementary content should contact the authors for assistance.

Brucella suis Infection in Cardiac Implantable Device of Man Exposed to Feral Swine Meat, Florida, USA

Appendix

Brucella Confirmation and Species Determination

The four suspected *Brucella* isolates were sent to the Spatial Epidemiology & Ecology Research Lab (SEER Lab) at the University of Florida Emerging Pathogens Institute, for confirmation, *Brucella* species identification, and whole genome sequencing. All procedures were carried out inside a BSL-3 laboratory at Emerging Pathogens Institute. DNA was extracted from all four live cultures. using the DNeasy UltraClean Microbial Kit (Qiagen, 12224–50) according to the manufacturer’s instructions. Final DNA eluate was sterilized by passing through a 0.22-micron filter (Corning Costar, 8160) and DNA was quantified using a Qubit 3 fluorometer and the Qubit dsDNA BR Assay Kit (ThermoFisher, Q32850). We used a well-described single nucleotide polymorphism (SNP) PCR assay (*1*), which can test for the presence of *B. abortus*, *B. melitensis*, *B. ovis*, *B. suis*, *B. canis* and *B. neotomae*.

Simultaneously, cultures were tested for motility using both the API M medium (bioMérieux, 50120) and Motility Test Medium with triphenyltetrazolium chloride for visual enhancement of growth, (Hardy Diagnostics, Q11). Briefly, well isolated colonies from fresh cultures were used to stab the center of the medium with the depth of the inoculum being equal to 2/3 of the height of the medium. Tubes were incubated at 37°C for 24–48 hours.

Whole Genome Sequencing and Genome Analysis

Genomic DNA was isolated as described above. The genomic DNA was fragmented from all four isolates and the sequencing library was produced using the NEBNext Ultra II DNA

library prep kit (New England Biolabs, E6177). DNA library size and quality was assessed by 2% (w/v) agarose gel electophoresis followed by analysis using a Bioanalyzer High Sensitivity DNA Kit (Agilent, 5067–4626). Paired end sequencing was carried out on the Illumina MiSeq next generation sequencer at the University of Florida Emerging Pathogens Institute with a 600-cycle MiSeq Reagent Kit v3 (Illumina, MS-102–3003). Demultiplexed reads were assembled with UniCycler (*2*) running on the University of Florida Research Computing Galaxy instance (*3*). The de novo assembly was run through the pubMLST database (*4*) species identification tool which uses rDNA sequence to identify species.

MLVA-15

To further confirm species and relate this strain to the global diversity of *Brucella* spp., we perform and *in silico* Multiple Locus Variable Number Tandem Repeat Analysis (MLVA) using a well describe 15 marker system (*5*). The MLVAfinder python script (https://github.com/i2bc/MLVA_finder) was used to identify the MLVA alleles at each locus. The allele profile for the *B. suis* strain isolated in this work was used to visualize the relationship between the Florida isolate and the global diversity described by Le Fleche et al. (*5*) with a minimum spanning tree produced using goeBURST + Euclidean distance algorithm in Phyloviz (*6*).

Results

The previously developed Gopaul SNP assay (*1*) was used to identify the species of *Brucella* isolated (Appendix Figure 1). The SNP assay was first used to test colonies from the already growing chocolate agar plate received by SEER Lab. Panels A-F depict signal from the FAM channel (530 nm), which corresponds to the non-specific allele present in species other than the one being tested by that specific master mix. The sample in question, depicted in red, shows strong amplification in the FAM channel for all master mixes except for the master mix which detects *B. suis* (Appendix Figure 1, panel D). The FAM channel also shows very poor to non-existing signal coming from the positive control (blue) tested in each master mix. This positive control (blue) corresponds to strains that have that species-specific polymorphism and thus would preferentially amplify with the VIC-labeled probe as shown in Panels H-M. No

amplification could be detected in the VIC channel for the sample being tested in any of the *B. abortus*, *B. melitensis*, *B. ovis*, *B. canis* and *B. neotomae* SNP assays. However, strong amplification of the unknown *Brucella* sample (red) was observed in the *B. suis* PCR in the VIC channel. Thus, the sample does not possess the specific SNPs for any of the *Brucella* tested, except for *B. suis*.

Similarly, DNA from the rest of tested cultures consisting of (I) freshly streaked the chocolate agar plate, (II) Brucella agar plate derived from nutrient agar slant, (III) broth culture inoculated with one well isolated colony, and (IV) broth culture inoculated with multiple colonies from chocolate agar plate fully grown on date of receipt, also tested positive for *B. suis* (Appendix Figure 2). Specifically, unknown *Brucella* samples showed little to no amplification with the FAM-labeled probe (Appendix Figure 2, Panel D) but strong and early amplification with the species-specific VIC probe along with the *B. suis* positive control (Appendix Figure 2, Panel I).

Motility

Cultures were also tested for motility using both the API M medium and Motility Test Medium with TCC. More than a dozen colonies were tested. All inoculums grew but failed to show any positive motility or diffusion throughout the agar (Data not shown).

MLVA Results

The MLVA-15 repeats are reported in the Appendix Table. The relationship between this strain and global representatives is presented in Appendix Figure 3. These results further confirm the SNP-based identification of *B. suis,* most closely related to *B. suis* biovar 1 (genotype 49 in (*5*)) and also closely related to genotypes 47 and 48, *B. suis* biovar 1 isolates from the U.S. confirming this case was infected with *B. suis* biovar 1.

References

1. Gopaul KK, Koylass MS, Smith CJ, Whatmore AM. Rapid identification of *Brucella* isolates to the species level by real time PCR based single nucleotide polymorphism (SNP) analysis. BMC Microbiol. 2008;8:86. PubMed https://doi.org/10.1186/1471-2180-8-86

2. Wick RR, Judd LM, Gorrie CL, Holt KE. Unicycler: Resolving bacterial genome assemblies from short and long sequencing reads. PLOS Comput Biol. 2017;13:e1005595. PubMed https://doi.org/10.1371/journal.pcbi.1005595

3. Afgan E, Nekrutenko A, Grüning BA, Blankenberg D, Goecks J, Schatz MC, et al.; Galaxy Community. The Galaxy platform for accessible, reproducible and collaborative biomedical analyses: 2022 update. [published correction appears in Nucleic Acids Res. 2022 Aug 26;50(15):8999. doi: 10.1093/nar/gkac610]. Nucleic Acids Res. 2022;50(W1):W345–51. PubMed https://doi.org/10.1093/nar/gkac247

4. Jolley KA, Bray JE, Maiden MCJ. Open-access bacterial population genomics: BIGSdb software, the PubMLST.org website and their applications. [version 1; peer review: 2 approved]. Wellcome Open Res. 2018;3:124. PubMed https://doi.org/10.12688/wellcomeopenres.14826.1

5. Le Flèche P, Jacques I, Grayon M, Al Dahouk S, Bouchon P, Denoeud F, et al. Evaluation and selection of tandem repeat loci for a Brucella MLVA typing assay. BMC Microbiol. 2006;6:9. PubMed https://doi.org/10.1186/1471-2180-6-9

6. Francisco AP, Vaz C, Monteiro PT, Melo-Cristino J, Ramirez M, Carriço JA. PHYLOViZ: phylogenetic inference and data visualization for sequence based typing methods. BMC Bioinformatics. 2012;13:87. PubMed https://doi.org/10.1186/1471-2105-13-87

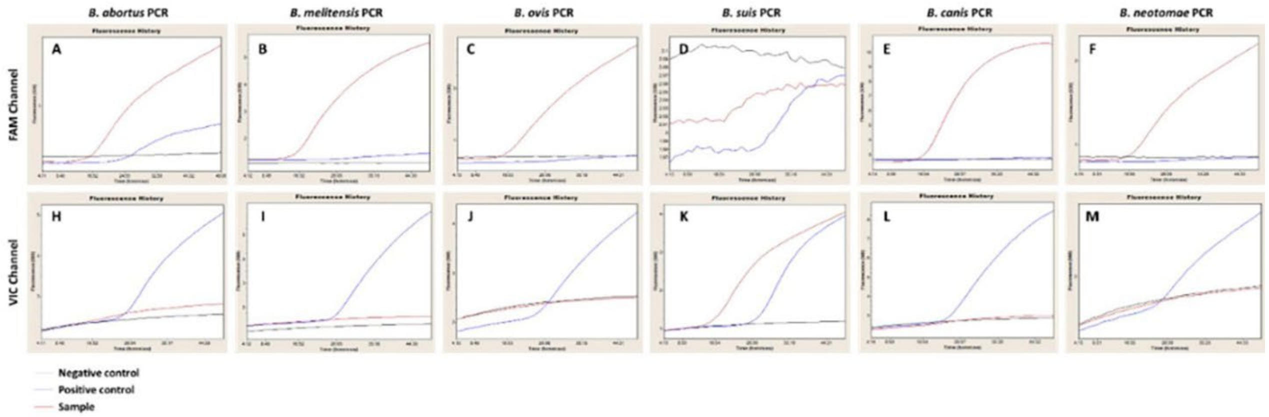

Appendix Figure 1. Gopaul assay for *Brucella* species identification. Each marker assay uses two probes labeled with either VIC or FAM. VIC-labeled probes preferentially bind to the species-specific polymorphism, whereas the FAM probe binds to the alternative allele present in the other species. Panels A-F depict signal from the FAM channel (530 nm), that is from the probe that binds to the non-specific SNP. Panels H-M depict signal from the VIC channel (560 nm), that is from the probe that preferentially binds to the species-specific SNP. Negative, positive, and unknown *Brucella* are depicted as black, blue, and red, respectively.

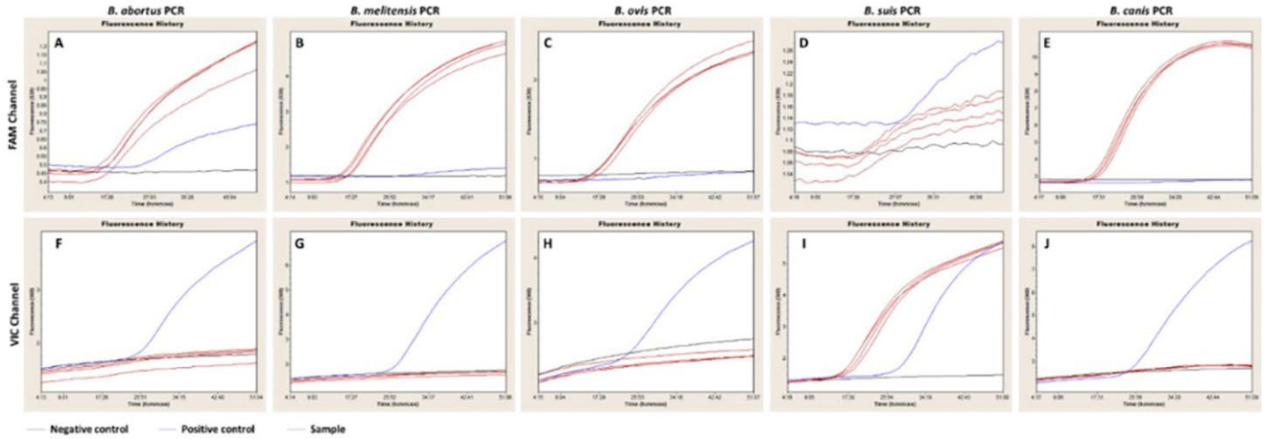

Appendix Figure 2. Gopaul assay for *Brucella* species identification. Unknown Brucella samples derived from (I) the chocolate agar plate that had been freshly streaked on the day it was received by SEER lab, (II) Brucella agar plate derived from nutrient agar slant, (III) broth culture inoculated with one well isolated colony from chocolate agar plate fully grown on date of receipt, and (IV) broth culture inoculated with multiple colonies from chocolate agar plate fully grown on date of receipt, were tested. Panels A-E depict signal from the FAM channel (530 nm) for detection of the probe that preferentially binds to the non-specific SNP. Panels F-J depict signal from the VIC channel (560 nm), for detection of probe that preferentially binds to the species-specific SNP. Negative, positive, and unknown *Brucella* are depicted as black, blue, and red, respectively.

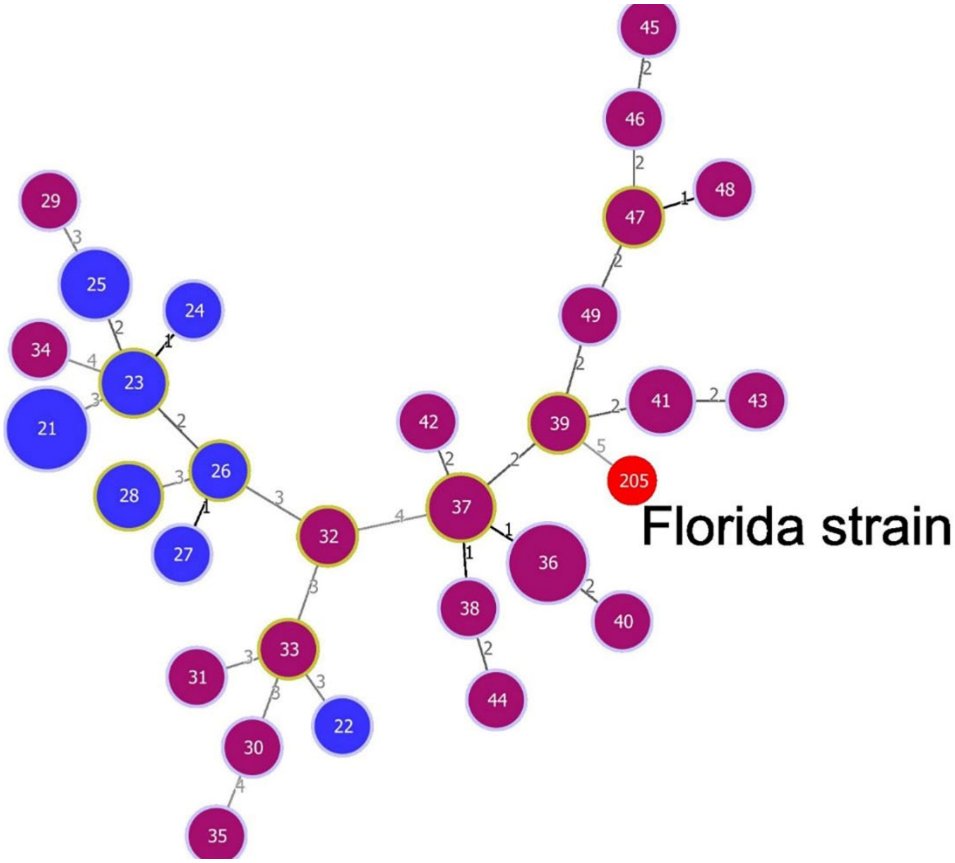

Appendix Figure 3. Minimum spanning tree relating the Florida strain (red dot #205) to global diversity based on the MLVA-15 genotyping system. Clonal complexes were analyzed at level 5 to enable facile comparison of the genotypes most closely related to the Florida *B. suis,* Blue dots are *Brucella canis* isolates and purple dots are *B. suis* strains present in the level 5 clonal complex. *B. suis* most closely related to the Florida *B. suis* are biovar 1. Numbers on circles are MLVA-15 genotypes as described by La Feche et al. (*6*). Numbers between dots indicate number of allele differences between genotypes.

Appendix Table. MLVA-15 repeats for the sample isolated from the case patient in a study of *Brucella suis* infection in cardiac implantable device of man exposed to feral swine meat, Florida, USA.

strain	Bruce06	Bruce08	Bruce11	Bruce12	Bruce42	Bruce43	Bruce45	Bruce55	Bruce18	Bruce19	Bruce21	Bruce04	Bruce07	Bruce09	Bruce16	Bruce30
B.suisVAFL	2	3	6	10	4	1	5	2	4	38	9.5	4	7	8	3	3
